# FuSe: a tool to move RNA-Seq analyses from chromosomal/gene loci to functional grouping of mRNA transcripts

**DOI:** 10.1093/bioinformatics/btaa735

**Published:** 2020-08-19

**Authors:** Rajinder Gupta, Yannick Schrooders, Marcha Verheijen, Adrian Roth, Jos Kleinjans, Florian Caiment

**Affiliations:** Department of Toxicogenomics, School of Oncology and Developmental Biology (GROW), Maastricht University, Maastricht, 6229ER, The Netherlands; Department of Toxicogenomics, School of Oncology and Developmental Biology (GROW), Maastricht University, Maastricht, 6229ER, The Netherlands; Department of Toxicogenomics, School of Oncology and Developmental Biology (GROW), Maastricht University, Maastricht, 6229ER, The Netherlands; Roche Pharmaceutical Research and Early Development, Roche Innovation Center Basel, Basel, CH-4070, Switzerland; Department of Toxicogenomics, School of Oncology and Developmental Biology (GROW), Maastricht University, Maastricht, 6229ER, The Netherlands; Department of Toxicogenomics, School of Oncology and Developmental Biology (GROW), Maastricht University, Maastricht, 6229ER, The Netherlands

## Abstract

**Summary:**

Typical RNA sequencing (RNA-Seq) analyses are performed either at the gene level by summing all reads from the same locus, assuming that all transcripts from a gene make a protein or at the transcript level, assuming that each transcript displays unique function. However, these assumptions are flawed, as a gene can code for different types of transcripts and different transcripts are capable of synthesizing similar, different or no protein. As a consequence, functional changes are not well illustrated by either gene or transcript analyses. We propose to improve RNA-Seq analyses by grouping the transcripts based on their similar functions. We developed FuSe to predict functional similarities using the primary and secondary structure of proteins. To estimate the likelihood of proteins with similar functions, FuSe computes two confidence scores: knowledge (KS) and discovery (DS) for protein pairs. Overlapping protein pairs exhibiting high confidence are grouped to form ‘similar function protein groups’ and expression is calculated for each functional group. The impact of using FuSe is demonstrated on *in vitro* cells exposed to paracetamol, which highlight genes responsible for cell adhesion and glycogen regulation which were earlier shown to be not differentially expressed with traditional analysis methods.

**Availability and implementation:**

The source code is available at https://github.com/rajinder4489/FuSe. Data for APAP exposure are available in the BioStudies database (http://www.ebi.ac.uk/biostudies) under accession numbers S-HECA143, S-HECA(158) and S-HECA139.

**Supplementary information:**

Supplementary data are available at *Bioinformatics* online.

## 1 Introduction

With the evolution of RNA sequencing (RNA-Seq), an immense amount of high-quality transcriptomics data has been generated; identifying and quantifying each gene transcript/isoform with high precision. Transcriptomics data are often studied to identify the changes in gene or transcript expression between different conditions and treatments. The ones exhibiting the highest perturbation at the lowest statistical error are then mapped to pathways and ontologies to illuminate the functional consequences of the alteration. However, the typical data analysis pipelines to assess gene expression from RNA-Seq dataset are not perfect. The expression level of a given gene is usually obtained by the summation of expression (read count) of all the different spliced variants (isoforms/transcripts) mapping the gene locus. These spliced variants are identified using the sequence identity to the genome and chromosomal locus. These isoforms can be protein coding (same or different proteins), non-coding and non-sense mediated decay. Considering the level of expression of a gene as the summation of all reads from these different types of isoforms is misrepresentative as it considers all of them as coding for the same protein.

Alternatively, analyzing RNA-Seq data at the level of isoform can also be performed by keeping an individual read count for every single transcript. Keeping each isoform separated would assume that there are no functional overlaps between different transcripts. Currently, most of the tools available to quantify RNA-Seq data like RSEM (Li and Dewey, 2011), StringTie (Pertea *et al.*, 2015), Sailfish (Patro *et al.*, 2014), Salmon (Patro *et al.*, 2015), Kallisto (Bray *et al.*, 2016) and HT-Seq (Anders *et al.*, 2015) along with others, focus chiefly on gene or transcript (isoform) expression. Cuffdiff (Trapnell, 2013), another read counts quantifying tool, groups different transcripts from the same transcription start site (TSS) to identify genes that are differentially regulated at the transcriptional or post-transcriptional level. None of these tools capture the functional similarity of the proteins coded by different transcripts. However, we know that closely related proteins are capable of exhibiting same functions and these proteins might be derived from different genes (paralogs) or from the same gene locus via alternative RNA splicing. Different histones (Mariño-Ramírez *et al.*, 2005)—HS1.1, HS1.2, HS1.3, HS1.4 and HS1.5 originating from same family of genes HIST1H1A–E, respectively, share functional similarities. Another well studied case is the ubiquitin-conjugating enzymes (Hegde, 2009)—E2D1, E2D2, E2D3 and E2D4 which originate from UBE2D1–4 genes, respectively.

There is no denying that functional overlap between proteins, derived from different genes and transcripts, exists and analyses focused on individual genes or transcripts would fail to translate to actual functional changes. A paradigm shift has to take place to move from gene/transcript-based to function-based analyses. To assess the importance of a given function, the actual amount of all proteins able to perform this function would need to be quantified. However, quantifying the proteins using the state-of-the-art proteomics technologies do not allow to have the exhaustive panel of expressed proteins (Pappireddi *et al.*, 2019) and hence, mRNA quantification data (using RNA-Seq) are a better alternative for establishing functional analyses. Indeed, all proteins’ primary structure can be predicted from their corresponding mRNA, a multitude of tools such as Translation Tool (Artimo *et al.*, 2012), EMBOSS Transeq (McWilliam *et al.*, 2013) or TranslatorX (Abascal *et al.*, 2010) are developed to accomplish this task using the knowledge of codon to an amino acid relationship, translation start or open reading frames. Moreover, considering the limitation of the proteomics, the quantified mRNA expression from the RNA-Seq experiments can provide a surrogate evaluation of protein expression at steady state (Liu *et al.*, 2016) and can be quantified using RNA to protein conversion factors otherwise (Koussounadis *et al.*, 2015).

Comparing the protein function and ontology profiles would provide the list of highly similar proteins; however, this would require a comprehensive protein-function–ontology knowledgebase which is not available. Around ∼20k SwissProt and ∼168k TrEMBL entries on Uniprot (date accessed: April 20, 2019) are available for humans (The_UniProt_Consortium, 2015). In the absence of such information, protein structure (tertiary and quaternary) seems a reliable option, as the function is chiefly defined by the structure. The lack of high-resolution structures, ∼3.5k proteins with <1.5 Å and ∼13.5k with 1.5–2.0 Å for humans on PDB (date accessed: April 20, 2019) (Berman *et al.*, 2000), and the unavailability of pure state protein structures due to protein stability poses a hindrance in defining the structure–function relationships. Different artificial intelligence and machine learning approaches have been employed to predict the protein structures (Moult *et al.*, 2018) but with limited precision and success because of multiple attraction and repulsion forces in action.

The only extensive high-quality information on the proteins available is the nucleotide sequence of their corresponding mRNAs. A comparison of these mRNAs is unsuited to find the similarity between them because of the presence and differences in intronic regions, (3′ or 5′) untranslated regions (UTRs) and coding sequence (CDS). Moreover, comparing the nucleotide sequences does not take into account the degeneracy (redundancy) of the genetic codon. Hence, the amino acid (primary protein) sequence of these proteins is taken for comparison. The primary sequence is readily available but in the case of unknown proteins, it can be achieved from the mRNA sequences. Their comparison can illustrate the local and global sequence identities but it is not enough to predict the functional similarity of the proteins. To supplement the comparison, data on the secondary structures are added to the comparison. A specific order of these structures gives rise to super-secondary structures and these can be used in envisaging the structural and functional features of the protein (Pelley, 2007).

Rather than defining gene expression that groups together the transcripts from the same chromosomal locus, we developed FuSe (functional grouping of transcripts for RNA-Seq analyses) with an aim to group protein coding transcripts based on the predicted similarity of their protein function. For this, we used the available primary structure of proteins and predicted the secondary, super-secondary structures, and protein families from it. For establishing the similarity from the protein primary sequence, BLAST+ (Camacho *et al.*, 2009) was used to identify sequence identity, coverage and gaps in the alignment. While sequence identity establishes the similarity between the proteins, the coverage provides information if the two sequences match globally or locally. The gaps further help in checking the presence of any insertions or deletions and hence provide information about the alignment continuity. Interpro is an ensemble of 14 different tools developed using state-of-the-art algorithms and knowledgebase to find and predict the domains, motifs and protein families (Mitchell *et al.*, 2018).

On the foundations of this information, two types of confidence scores, knowledge (KS) and discovery (DS), are calculated for all protein pairs. KS is stringent and predicts highly similar protein pairs whereas DS is lenient and predicts the proteins with local similarity as well. Based on the confidence score, ‘similar function protein groups’ (SFPGs) are formed from the overlapping protein pairs and are used for recalculating the RNA-Seq expression. To assess the approach and illustrate the changes in functional inferences between the chromosomal locus and function-based grouping, mRNA data from hepatic cell models exposed to acetaminophen (APAP or paracetamol) were used.

## 2 Materials and methods

All the protein coding transcripts for human were downloaded from Ensembl (Homo_sapiens.GRCh38.pep.all.fa) (Frankish *et al.*, 2018). For the workflow of FuSe, refer Supplementary Figure S1.

### 2.1 Data preparation

To find the similarity between the protein sequences, BLAST+ (v.2.8.0) was used and data was generated in tabular format 6 of BLAST+. Then, to find and predict the presence of structural and functional domains in the proteins, Interpro (v.5.31–70.0) was used. The data were obtained in the ‘.tsv’ format.

The output from Interpro was a list of functional and structural domains obtained from various tools embedded in Interpro. Using in-house developed scripts, for each protein, these domains are first ordered based on their position on the amino acid sequence per tool. These ordered domains were then compared for similarities between the protein pairs. The similarity between the ordered domains was labeled for each tool per protein pair as STONM (same type, order and number of motifs), STNM (same type and number of motifs), STM (same type of motifs) and NM (no match) (Supplementary Fig. S2). STONM defines the highest level of similarity. Another term, NP (not present) was assigned to cases where there was no prediction by an Interpro tool for at least one of the proteins in the given protein pair. Each protein pair will have one term (STONM, STNM, STM, NM or NP) for each of the 14 tools and, from this information, a protein–protein domain profile per tool is obtained.

### 2.2 Protein pair confidence scores

From the BLAST+ and protein–protein domain profile comparison, the protein pair confidence scores were calculated using a scoring scheme (Supplementary Methods). Two types of confidence scores, DS and KS, were calculated; succinct and expanded equations are as follows:

Succinct equations:

(1) $$\mathrm{DS}=\frac{\left(\mathrm{AIS}+\mathrm{ACS}+\;\mathrm{AGS}\right)*100}{\mathrm{Max}\;\mathrm{AIS}}$$

 

(2) $$\mathrm{KS}=\mathrm{AIS}+\mathrm{ACS}+\;\mathrm{AGS}+\frac{\mathrm{ITCS}*100}{\mathrm{Max}\;\mathrm{ITCS}}$$

where AIS, alignment identity score; ACS, alignment coverage score; AGS, alignment gap score; ITCS, sum of Interpro tools’ comparison score.

Expanded equations:

Discovery score (DS)= % Identity*Identity score100+ ∑i=12100-seq lengthalignment length*100i* Coverage score100+Number of gaps*Gap score100* 100Identity score(3)

 

Knowledge score (KS)=(% Identity*Identity score100+∑i=12100-seq lengthalignment length*100i* Coverage score100+Number of gaps*Gap score100+∑j=114if comparison=STONMSTNMSTM|NM, Interpro tool scorejelse if comparison= NP, skip)* 100∑j=114if comparison=STONMSTNMSTM|NM,  Max Interpro tool score jelse if comparison=NP, skip(4)

The DS relies only on the sequence similarity attributes obtained from sequence alignment such as identity, coverage and gaps. While identity and coverage score have a positive value, they illustrate the similarity between the protein pair, the Gap score has a negative value and demonstrates their dissimilarity. The score obtained is then normalized to 100 using the maximum possible alignment identity score. In the case of KS, the final score is a result of sequence similarity attributes and ordered secondary structure similarity given as STONM, STNM, STM, NM or NP. To avoid penalizing protein with missing prediction information for one (or more) of the 14 Interpro tools (Supplementary Fig. S3), we then normalize the ITCS to the maximum possible ITCS.

### 2.3 Similar function protein groups

Confidence scores were calculated from all possible protein pairs as described in the previous step. To identify the proteins which are similar in function, a confidence score cutoff (CSC) was used with a default value of KS ≥ 95. The CSC can be any positive integer ≤100. A lower CSC would result in the formation of SFPG with false positives. It is important to establish here that a given transcript can be a member of one or more SFPGs, as it can have certain a sufficient amount of similarity (above the assigned DS or KS threshold) with transcripts belonging to different SFPGs.

### 2.4 Calculating SFPG expression

This step is divided into two parts: first the normalization of the raw reads followed by the calculation of the SFPG expression. For the normalization, the raw read counts of the transcripts and their effective length were used as calculated by RSEM.

*Normalization*: For the calculation of the SFPG expression, the read counts need to be both in-sample and across-samples normalized. While Fragments Per Kilobase of transcript per Million (FPKM) is in-sample normalized, it is not comparable across different samples. Additionally, while the normalized read counts generated using one of state-of-the-art method (such as DESeq2 or edgeR) focuses on normalizing for library depth and genes densities to compare the same transcript among different treatment groups, it does not allow the absolute comparison of transcripts of a different length. To address these concerns, we combined these two normalization approaches and generated expression which is in-sample and across-samples normalized. We first normalized the raw read counts for the transcripts using the DESeq2 default normalization method and then, using the effective length of the transcripts as given by the RSEM, converted these normalized read count into FPKM (cf. normalized_fpkm module on FuSe’s GitHub repository).

*Expression of SFPGs*: Using the normalized FPKM and SFPGs formed in step 3, SFPG expression is then calculated. The calculation of the SFPG expression can be achieved using one of two proposed approaches available in FuSe: (i) equal distribution (ED) or (ii) group size distribution (GD) available under ‘recal_expression’ module. In the case of equal distribution, the expression of the transcript is equally divided between all the SFPGs of which it is a member [Equation (5)], thus giving equal importance and weight to all individual members of all SFPG. For the group size distribution, the expression of the SFPGs is based on the number of members present in each SFPG [Equation (6)] (Supplementary Fig. S4). If the equal distribution is used, each function, as defined by an SFPG, is given equal importance whereas group size distribution is based on the concept of genetic redundancy (Nowak *et al.*, 1997), giving higher importance to bigger groups. Group size distribution is illustrated in Supplementary Figure S5.

(5) $$\mathrm{Equal}\;\mathrm{distribution}\;=\;\sum_{i=1}^{\mathrm{No}.\;\mathrm{of}\;\mathrm{members}\;\mathrm{in}\;\mathrm{SFPG}}\frac{\mathrm{Normalized}\;\mathrm{FPKM}\;\mathrm{of}\;{\mathrm{member}}_{i}}{\mathrm{No}\;\mathrm{of}\;\mathrm{SFPGs}\;\mathrm{for}\;{\mathrm{member}}_{i}}$$

 

(6) Group size distribution = ∑i=1No.ofmembersinSFPGNo of members in current groupNo of members in all groups of memberi*memberi normalized FPKM

Using the GRCh38 for humans from Ensembl, we have created the data object (bi_do; BLAST Interpro data object) which can be used for further analyses. For using the future updates to Ensembl protein sequences, BLAST+ or/and Interpro, create a new bi_do using the steps mentioned on FuSe’s Github repository. The data object provided is generated using the protein coding transcripts only; however, if the user intends, other types of transcripts can also be used for instance non-sense mediated decay, polymorphic pseudogene, non-stop decay, etc. refer Readme for FuSe’s repository on GitHub.

### 2.5 Assessment of FuSe

To illustrate the significance of using FuSe, we used a RNA-Seq dataset obtained from a three-dimensional human hepatic cell model (Primary Human Hepatocytes + Kuepfer cells Spheroids from InSphero^®^) exposed to APAP. Ribo-depleted libraries were generated from these cell models and sequenced on an Illumina Hiseq2000 (Supplementary Methods) at an average of 41.3 million reads per sample in 100 bp paired-end (Supplementary Fig. S6). There were four sets of samples: control untreated (ConUNTR), control exposed to DMSO (ConDMSO), exposed to therapeutic dose (Ther) and exposed to toxic dose (Tox). ConUNTR and Ther had five time points: 0, 2, 8, 24 and 72 h, and ConDMSO and Tox had four time points: 2, 8, 24 and 72 h. Each time point had three replicates, totaling 54 samples. The therapeutic dose was calculated based on physiologically based pharmacokinetic modeling using human kinetic data, and the toxic dose was obtained from IC20 (Kuepfer *et al.*, 2018). Data are available in the BioStudies database (http://www.ebi.ac.uk/biostudies) under accession numbers S-HECA143, S-HECA(158), S-HECA139 (APAP link for reviewers: https://www.ebi.ac.uk/biostudies/studies/S-HECA143?key=31dad9db-da50-47a6-b16e-e9108e935f43). Reads above Q30 for ConUNTR, ConDMSO, Ther and Tox samples constituted 85.19%, 88.43%, 94.5% and 94.2% of all reads, respectively. FPKM for transcripts was calculated using RSEM and from its expression for SFPGs was calculated using FuSe. Principal component analysis (PCA) and hierarchical clustering were done for top 500 expressed transcripts using R packages: prcomp() and hclust(), respectively, for isoform FPKM and recalculated SFPG expression to compare them. Then differentially expressed transcripts (DETs) were evaluated using Anova package in R for all dose versus control samples: ConUNTR versus The, ConUNTR versus Tox, ConDMSO versus The, ConDMSO versus Tox and Ther versus Tox samples. Significant cutoff was set to *P*-value < 0.01 and |log2FC|>1. Changes in the DETs between the original FPKM and recalculated expression were also established.

## 3 Results

**Fig. 1.**
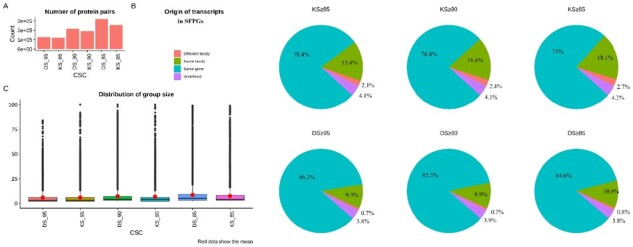
Characterization of protein pairs and SFPGs formed for KS and DS ≥85, 90 and 95. (**A**) The number of protein pairs and (**B**) the origin of the transcripts that make the protein pairs. These can be derived from the same gene, same gene family, different gene family or unknown; it is determined based on the gene names. The genes, which are assigned numeric identifiers, sometimes with version numbers, are categorized as undefined. (**C**) The distribution of the number of groups. The mean (given as red dots) can be seen increasing as the CSC is decreased but the median stays comparatively lower

In order to move from a loci-based transcriptomics analysis to a function-based analysis, we first need to identify all protein coding transcripts from the human genome. For this, a total of ∼107k proteins were retrieved from Ensembl, of which 719 proteins were discarded which originated from transcripts annotated as non-sense mediated decay, polymorphic pseudogene, T-cell receptor genes and immunoglobulin genes. From the remaining transcripts, protein pairs were formed and two confidence scores (KS and DS) were calculated to estimate the likelihood of similar functions. Taken individually, both scores are used to make the protein pairs and a CSC (here used: 85, 90 and 95 for both KS and DS) is introduced to discard the protein pairs with low similarity. While both DS and KS show a steep increase in the number of pairs with the lowering of CSC, a steep increase for DS can be seen (Fig. 1A). The protein pairs can be divided into four categories depending on the origin of the transcripts, namely, same gene, same gene family, different gene or undefined (Fig. 1B). A considerable amount of protein pairs originated from the same or different gene families at KS and DS ≥95. Moreover, a surge can be witnessed with decreasing CSC.

With the protein pairs at different CSCs, SFPGs are formed and a similar trend of increase in the group size (Fig. 1C), the sum of group sizes per CSC and maximum group size for an SFPG (Supplementary Fig. S5A) with decreasing CSC can be seen. The median for the group size per CSC remains low (Supplementary Fig. S5B), implying that most groups are small. The increase in group size for the biggest group per CSC was also apparent (Supplementary Fig. S5C). For KS and DS at ≥95, the largest group size was 84 and 111 which rose to 216 and 229 at ≥85, respectively.

**Fig. 2.**
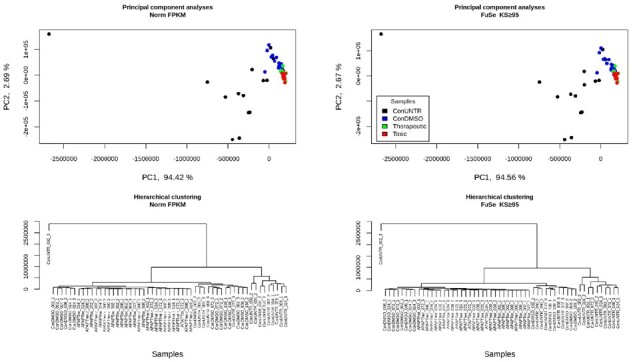
The PCA bi-plots and clustering of the original FPKM and recalculated expression at KS ≥ 95. Little to negligible effect was observed on PCA plots. From the clustering, it can be seen that the biggest source of variation is dose and is conserved after the recalculation. The samples are named as ‘treatment_timepoint_replicate’

To evaluate the impact of SFPG on biological interpretation, we applied FuSe on an *in vitro* transcriptomics dataset obtained from a hepatic cell model exposed to different doses (therapeutic and toxic) of APAP for variable time duration and corresponding controls (untreated and DMSO). The dataset was analyzed by locus-based and our function-based method. For this, SFPGs at KS ≥ 95 were taken to particularly consider the highly similar protein pairs predicted using maximum available knowledge. The expression for the SFPGs was calculated from the normalized FPKM using both methods, namely, ‘*ED*’ and ‘*GD*’ (cf. Section 2.4). Differences in the recalculated expression from *ED* and *GD* are discussed in Supplementary Results and Fig. S7. The primary analyses shown in this paper makes use of the recalculated expression obtained using the ‘*GD*’ method to give a higher importance to bigger SFPGs, implying that important biological processes are conserved. The term ‘recalculated expression’ from here on would mean the recalculated expression from ‘*GD*’. The normalized FPKM and recalculated expression (GD) from FuSe were then compared. PCA bi-plots and clustering (Fig. 2 and Supplementary Fig. S8A–E) were done to show that FuSe preserves the inter-sample variation and global profile of the samples. The highest source of variation between the samples was dose and it was conserved.

**Fig. 3.**
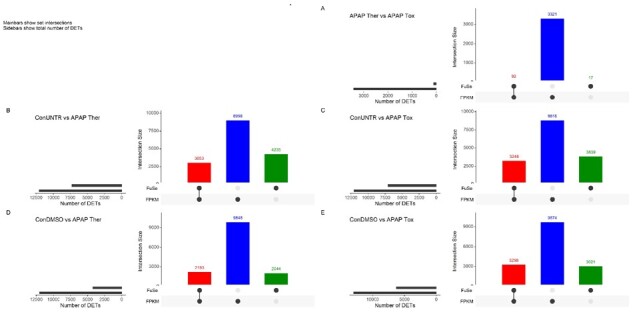
Comparison of DETs. Change in DETs (basemean > 10 and *P*val < 0.01 and |log2FC|> 1) obtained from FPKM and FuSe expression. The DETs were calculated using DESeq2 and the plots were made using UpSetR package (Conway *et al.*, 2017) in R. (**A**) Ther versus Tox, (**B**) ConUNTR versus Ther, (**C**) ConUNTR versus Tox, (**D**) ConDMSO versus Ther and (**E**) ConDMSO versus Tox. The red bars show overlapping DETs, blue and green bars show exclusive DETs from original FPKM and recalculated expression, respectively

Furthermore, DETs were calculated for the FPKM and recalculated expression obtained using FuSe, individually for all control versus dose samples. Lesser number of DETs were observed after applying FuSe (Fig. 3 and Supplementary Table S1). We also observed many transcripts that were significantly differentially expressed in FPKM but non-significant after using FuSe and vice versa (Fig. 3, blue bars and green bars, respectively). With loci-based analyses, the differences were computed at the transcript level while using FuSe the changes at the functional level were captured. Using FuSe, the expression levels were correctly quantified as a result of the expression of other similar function proteins. In the case of ConUNTR versus Tox and ConDMSO versus Tox, this is illustrated by protein coding transcripts from many genes responsible in cell adhesion and tight junction such as *PKP2-201* (Plakophilin-2), *CHCHD3-203* (MICOS complex subunit), *CHCHD3-201* (MICOS complex subunit MIC19), *IMMT-205* (MICOS complex subunit MIC60), *AGRN-201* (Agrin), *WDR1-205* (WD repeat-containing protein 1), *CTNNA1-243* (Catenin alpha-1), *ZBTB33-202* (Transcriptional regulator Kaiso), *ASPH-201* and *ASPH-207* (Aspartyl/asparaginyl beta-hydroxylase). All these transcripts, not considered differentially expressed by the standard analysis method, were found significantly affected by a toxic dose of APAP after applying FuSe.

**Fig. 4.**
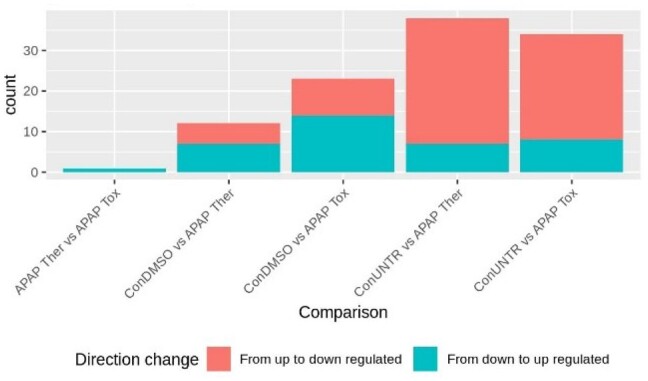
Number of DETs changing the direction of regulation. Illustrates the number of transcripts for which the change in the direction of regulation of the DETs (basemean > 10 and *P*val < 0.01 and |log2FC|>1) was observed. The comparison was made for DETs from standard FPKM and recalculated FPKM analyzed using FuSe

**Fig. 5.**
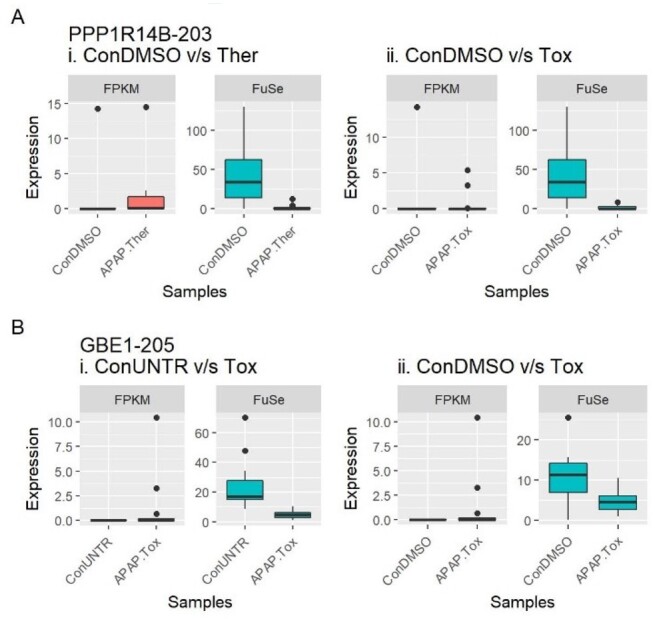
Effect of using FuSe. Several DETs changed their direction of regulation (up- or downregulated); here illustrated using two cases. (**A**) *PPP1R14B-203* was upregulated with the standard method but, after the functional grouping-based analyses, it was shown to be downregulated for (i) ConDMSO versus APAP Ther and (ii) ConDMSO versus APAP Tox. (**B**) Similarly in the case of *GBE1-205*, for (i) ConUNTR versus APAP Tox and (ii) ConDMSO versus APAP Tox, it was exhibited as downregulated regulated after applying FuSe

More importantly, some transcripts displaying a significant regulation (up or down) using the conventional analysis method were found to be significantly regulated in the opposite direction after using FuSe (Fig. 4 and Supplementary Fig. S9). The highest number of switches was seen for ConUNTR versus Ther. A total of 79 unique transcripts changed their direction of regulation (from upregulated to downregulated and vice versa), and 3727 changed from differentially expressed to not differentially expressed (and vice versa) across all comparisons. As an example, *PPP1R14B-203* (Protein phosphatase 1 regulatory subunit 14B) and *GBE1-205* (1,4-alpha-glucan-branching enzyme), which are protein coding transcripts, demonstrated a change in the direction of regulation (Fig. 5). While for *PPP1R14B-203*, the change could be seen for ConDMSO versus Ther and ConDMSO versus Tox, for GBE1-205, the switch was witnessed for ConUNTR versus Tox and ConDMSO versus Tox.

**Fig. 6.**
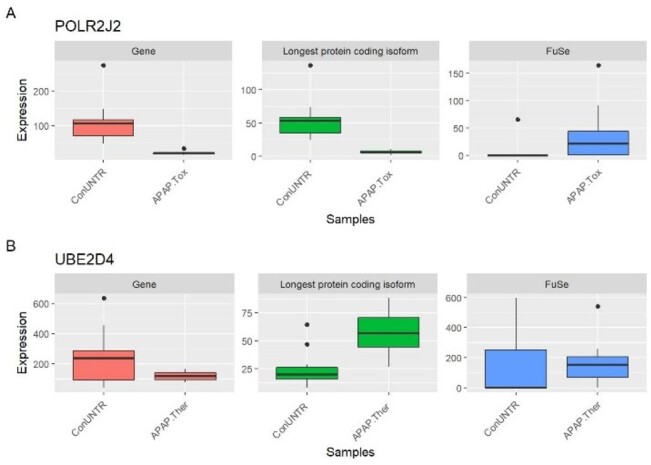
Expression of the gene, the longest protein coding isoform and SFPG from FuSe. The typical gene expression analysis is obtained by summing all the transcripts (protein coding and non-coding) from the locus whereas in our proposed functional grouping, only the transcripts making the same protein are grouped and expression is calculated for the SFPG. The longest protein coding isoform from the gene was chosen as a representative of the gene to be compared directly with SFPG. (**A**) *POLR2J2*; the gene expression is the result of two protein coding transcripts whereas the SFPG is constituted by four similar protein coding transcripts. (**B**) *UBE2D4*; the gene expression is the result of 11 transcripts (two protein coding and nine non-coding transcripts) whereas the SFPG is constituted by 18 similar protein coding transcripts. Similar function proteins can arise from the same or different genes

FuSe also demonstrated how gene expression-based analyses sometimes lead to incorrect results. As there are multiple different protein coding transcripts for a gene, to compare the expression of the gene to the transcripts, we selected the longest protein coding transcripts for the comparison. Moreover, for the APAP study, there were ∼59% genes where the longest protein coding transcript was the highest expressed transcript; making them a suitable candidate for the comparison. The differences between the gene and SFPG expression can be illustrated here using two cases, *POLR2J2* (RNA polymerase II subunit J2) from ConUNTR versus Tox and *UBE2D4* (ubiquitin-conjugating enzyme E2 D4) from ConUNTR versus Ther (Fig. 6). While the gene expression of *POLR2J2* is contributed by two transcripts (both protein coding), the expression of SFPG (*POLR2J2-202*) is attributed by four similar protein coding transcripts (KS = 98.02). The expression of the longest protein coding isoform follows the pattern of the gene expression; however, the SFPG expression shows the opposite. For *UBE2D4*, the gene expression comprises of expression of 11 transcripts (one retained intron, one processed transcript, seven non-sense mediated decay and two protein coding), the SFPG expression of the longest protein coding transcript from the gene (*UBE2D4-201*) is contributed by 18 similar protein coding transcripts (KS = 96.05). The longest protein coding transcript follows the pattern of SFPG though the magnitude is much higher as illustrated by SFPG.

## 4 Discussion

Biological research aims to find the functional properties and changes in the biological system, which makes us question the very fabric of our current RNA-Seq analyses strategies that focuses on changes in individual genes or transcripts. While the information of each gene or transcript are informative; however, at the system level, contribution by each element should be viewed in terms of functional change. Elevating the analyses of the RNA-Seq to the functional level will increase our understanding of the biological systems and their underlying processes. Due to the limitations in quantifying the whole panel of expressed proteins and limited knowledge of protein tertiary and quaternary structure, and functions identifying the proteins having similar functions is challenging. Here, we focused on the primary and secondary structure of the proteins to establish their functional similarity.

The hypothesis that similar primary sequence and secondary structures make the proteins possessing similar functional properties can be challenged at various levels; however, it is an acceptable hypothesis (Gong and Rose, 2005) and holds true for most cases as shown by studies of homologs and paralogs (Jensen, 2001). Protein trees calculated from sequence similarity often have the same topologies as those calculated from structural similarity. While some cases might be overlooked for instance a point mutation, which can result in a different conformation of the protein, they will not have a significant influence on the sequence alignment and secondary structure prediction (if not present in the prediction region). Moreover, other external factors such as molecular crowding or macro-molecular environment cannot be accounted for while taking into account only the primary and secondary structures.

Even though primary and secondary structures exhibit some limitations to predict similar proteins, they allow us to group similar function proteins and form SFPGs. FuSe uses amino acid sequences of the proteins thus eliminating all non-coding transcripts from the analyses. While many non-coding transcripts have been associated with a specific function, not enough data are available at this stage to allow a generic *in silico* grouping of non-coding RNA sharing a similar biological function. Moreover, the transcripts annotated as non-sense mediated decay, polymorphic pseudogene, T-cell receptor genes and immunoglobulin genes were removed from the formation of SFPGs. Non-sense mediated decay transcripts were removed because they are destined to be decayed before entering the ribosomal machinery for protein synthesis. In the case of the polymorphic pseudogenes, they have lost their functional properties over evolution and may result in non-functional proteins. T-cell receptor and immunoglobulin genes are very selective for their targets and even if they share high similarity among them, they have different affinities for their targets and hence cannot be considered as a similar functional entity. Even though these transcript types are removed from the creation of SFPGs, they are retained with their original expression in the recalculated expression.

The SFPGs are, hence, formed of only the protein coding transcripts. The similarity of the proteins is established using the two score types: DS and KS. DS makes an over-prediction because it relies only on the amino acid sequence, providing little information on the final structure and hence function(s) of the protein. KS is more conservative and takes into account other available knowledge to establish similarity. The lenient nature of DS makes it a powerful tool to find novel protein pairs whereas KS, being stringent, under-estimates and limits the ballooning of the groups by keeping only highly similar proteins together. DS can be used in finding the local similarities in proteins and thus allows the discovery of a subset of common functions between two proteins whereas KS thrives for global functional similarity.

It is worth noting here that an SFPG is made for each protein coding transcript that has other similar protein coding transcripts, in order to preserve the specialized protein functions. It resulted in redundancy, as some transcripts can be a member of multiple SFPGs and these SFPGs might then be semi- or fully overlapping. If such SFPGs were merged, it would result in the formation of false-positive SFPGs (decreased specificity) and the consecutive loss of some specialized protein functions. As some transcripts were shared between many groups, for the calculation of expression of the SFPGs, two methods are made available. First, equally dividing the expression of member transcripts between all the overlapping SFPGs and the other where the expression is divided between the SFPGs based on the group size of each SFPG, giving higher expression to bigger groups. While the first method is conservative giving equal importance to all SFPGs, the second is biased toward the bigger SFPGs, establishing that the important functions are more preserved. The quantification of the SFPG expression using FuSe requires in-sample normalized data because it needs to compare the transcripts within the sample for its calculation. Moreover, the expression should also be normalized across samples to compare it across samples. We calculated the FPKM from the normalized expression which was obtained using DESeq2. As a consequence, SFPGs are advised to be studied in relative analysis comparing a given SFPG between two biological conditions rather than for evaluating their absolute expression level among the different SFPGs.

The results from the comparison of FPKM and expression of SFPGs using FuSe from the hepatic cell model established that the changes in the expression of the transcripts acquired using FuSe do not change the overall look of the samples, though the changes at the level of SFPGs were apparent and pointed toward different functional inferences. For instance, genes responsible for cell adhesion and tight junctions were initially shown to be not differentially expressed. However, the application of FuSe completely changes the biological interpretation of this signal, confirming the documented APAP effects (Blair *et al.*, 1989; Gamal *et al.*, 2017).

There were also cases of transcripts that were differentially expressed in the opposite direction after correction using FuSe, e.g. *PPP1R14B-203* (ConDMSO versus Ther) and *GBE1-205* (ConUNTR versus Tox) (Fig. 4). The correction by FuSe reversed the direction of perturbation and hence completely changed the inferences drawn from the results. *PPP1R14B* is responsible for inhibition of *PPP1CA*, which is involved in different processes such as cell division, regulation of glycogen metabolism, muscle contractility and protein synthesis via dephosphorylation (Mi *et al.*, 2007; Song *et al.*, 2015). For the therapeutic dose of APAP, the upregulation of *PPP1R14B* as shown by FPKM-based differential expression would imply all these processes to be inhibited. Similarly, *GBE1* was shown to be upregulated under APAP toxic dose, implying that the glycogen accumulation in the liver has increased. However, APAP is known to induce glycogen depletion and is considered as one of the early biomarkers of acetaminophen-induced hepatotoxicity (Gautam *et al.*, 2012). Using FuSe, the GBE1 function is shown to be downregulated. The use of SPFGs also demonstrated why studying gene expression to attain differentially expressed genes can be miss leading, as in the case of *UBE2D4* (gene expression: downregulated; SFPG expression: upregulated) and *POLR2J2* (gene expression: upregulated; SFPG expression: downregulated). *UBE2D4* is involved in ubiquitination (David *et al.*, 2010) and *POLR2J2* is an important component of RNA polymerase II. The downregulation of *POLR2J2* would result in a decrease in transcription while the upregulation of *UBE2D4* implies more ubiquitination leading to increased protein degradation. This suggests a decrease in protein levels in the cell, and hence, the disruption of cell processes, which is consistent with the knowledge on APAP overdose.

FuSe showed how moving from loci-based to function-based analyses changed the inferences derived from the RNA-Seq data. It illustrated functional changes that could not be captured using the conventional RNA-data analyses. Moreover, FuSe is forward compatible and new data that will be available in the future for transcripts’ protein sequences and secondary structures can be integrated into the analysis by following the steps mentioned for ‘creating your own BLAST Interpro data object’ under Section 2. Lastly, the transcripts coding for the same protein and originating from overlapping chromosomal locations but annotated to different genes have to be studied further to understand the processes and signals responsible for guiding different genes to make similar proteins. In the future, we will look to fine-tune the calculation of CSC using other inherent features of the proteins such as molecular weight, charge, electrophoretic properties, active sites or hydrophobic-hydrophilic properties. Furthermore, to integrate all these features, new pipelines and algorithms will be investigated and developed.

## Supplementary Material

btaa735_Supplementary_Data
