# Supplementary material for: FuSe: a tool to move RNA-Seq analyses from chromosomal/gene loci to functional grouping of mRNA transcripts
Source: Bioinformatics. 2020 Aug 19;37(3):375–81. doi: 10.1093/bioinformatics/btaa735 (PMC8058771; doi:10.1093/bioinformatics/btaa735)
Supplement: btaa735_Supplementary_Data [file btaa735_supplementary_data.zip › Suppl.docx]

**Supplementary methods**

1. Sample and data preparation
2. RNA sequencing

Total RNA was isolated using Qiagen AllPrep DNA/RNA/miRNA Universal Kit (Cat #80,224). The sample was depleted of ribosomal RNA using the Illumina RiboZero Gold kit (Cat #MRZG12324) and prepared for sequencing using Lexogen SENSE total RNA library preparation kit (Cat #009.96). After library preparation, the samples were sequenced on the HiSeq2500 (100bp paired-end). A pool of all hepatic samples was sequenced on all 8 lanes of a flow cell.

1. Data pre-processing

The quality of the RNA-Seq raw data (fastq files) was analyzed using the Fastqc (version 0.10.1) [[1](#_ENREF_1)] , fastp [[2](#_ENREF_2)], and MultiQC [[3](#_ENREF_3)] and after considering the quality of the sequences, tails of the sequences were trimmed of the bad quality of the sequences (twelve nucleotides) using Trimmomatic (version 0.33) [[4](#_ENREF_4)]. The sequences were mapped onto the Ensembl [[5](#_ENREF_5)] human genome (version 84) using Bowtie2 (version 2.2.6) [[6](#_ENREF_6)], and gene and isoform (transcript) counts were calculated using RSEM (version 1.2.28) [[7](#_ENREF_7)].

**Supplementary protocol**

1. Calculation of expression for SFPGs using number of members distribution

SFPG expression is calculated using the FPKM of each protein (transcript) member. The calculation is dependent on three factors, for say SFPG ‘X’:

1. number of members in SFPG ‘X’
2. FPKM of each member in SFPG ‘X’
3. number of members in SFPG of each member in SFPG ‘X’

It is based on the assumption that higher the number of members in a functional group (SFPG), the more important is the function. The SFPG having more members are then attributed more expression count from each member; Suppl. Figure 3 illustrates a dummy case of SFPG expression calculation.


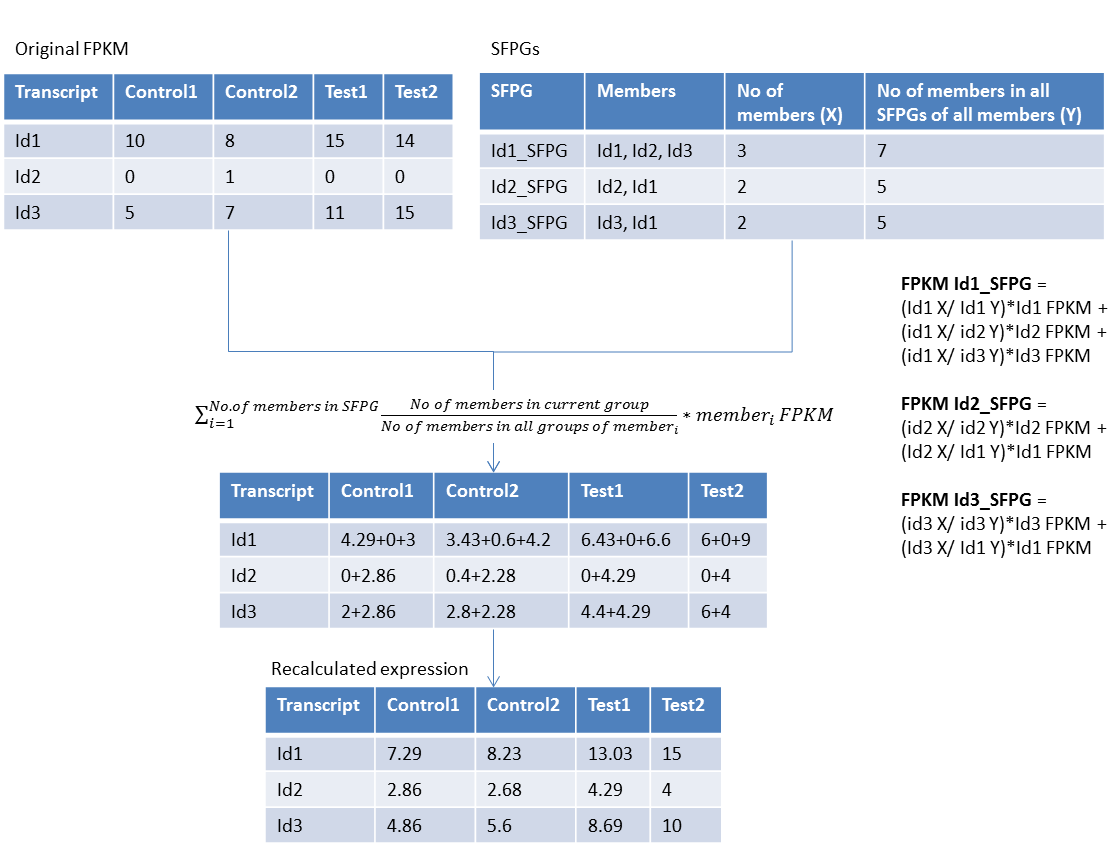


Suppl. Figure 4: A dummy example to illustrate the calculation of expression for the SFPGs; the calculations are done based on equation 3.

1. Scoring scheme

A scoring scheme is a set of scores given to each attribute in the comparison. The features taken from primary structure (amino acid sequence alignment) comparison are identity, coverage (differences in coverage between the two sequences), and gaps. Identity has a positive score of 30 whereas coverage and gaps have a negative score of -5 and -10 respectively. For comparing the secondary, super secondary and protein families Interpro results generated using 14 tools: CDD [[8](#_ENREF_8)], Coils, Gene3D [[9](#_ENREF_9)], Hamap [[10](#_ENREF_10)] MobiDBLite [[11](#_ENREF_11)] Pfam [[12](#_ENREF_12)], PIRSF [[13](#_ENREF_13)], PRINTS [[14](#_ENREF_14)], ProSitePatterns [[15](#_ENREF_15)], ProSiteProfiles [[15](#_ENREF_15)], SFLD [[16](#_ENREF_16)], SMART [[17](#_ENREF_17)], SUPERFAMILY [[18](#_ENREF_18)], and TIGRFAM [[19](#_ENREF_19)] were taken. The comparison results gave one of the listed annotations to each pair: STONM, STNM, STM, and NM; with a score of 5, 2.5, 1, and -2.5, respectively. If the comparison result is NP, no score is awarded. The cases of NP are addressed at the time of confidence score calculations, as given in the original paper.

**Supplementary Results**

SFPG expression using equal distribution (ED) versus group size distribution (GD)

The SFPGs obtained using KS≥95 were used to illustrate the differences in expression calculation from ED and GD. For calculating SFPG expression, equal distribution (ED) gives the same importance to all SFPGs irrespective of their size (number of members) while group size distribution (GD) provides more importance to bigger groups and aims at focusing on preserved functions. In Suppl. Figure 7, the differences in the DETs obtained from the recalculated expression using both of these recalculation approaches are displayed. While a large overlap between the two approaches can be observed, distinct transcripts in both ED and GD can also be noticed. The total number of DETs was found to be very similar for both methods. For Instance, APAP Ther reported 866 DETs with ED mode versus 876 in GD, and ConDMSO versus APAP Ther shown 10402 and 10394 DETs, respectively. This observation is consistent over all tested conditions (Suppl. Figure 7). Across all comparisons, a total of 267 and 287 unique differentially expressed transcripts were predicted by ED and GD methods, respectively. An over-representation analysis of these unique DETs did not report any over-represented molecular function, cellular component, or biological process. The group sizes (SFPGs at KS≥95) for the distinct transcripts varied from 2 to 25 for ED (mean: 4.95, median: 4) and 2 to 23 for GD (mean: 5.66, median: 5).


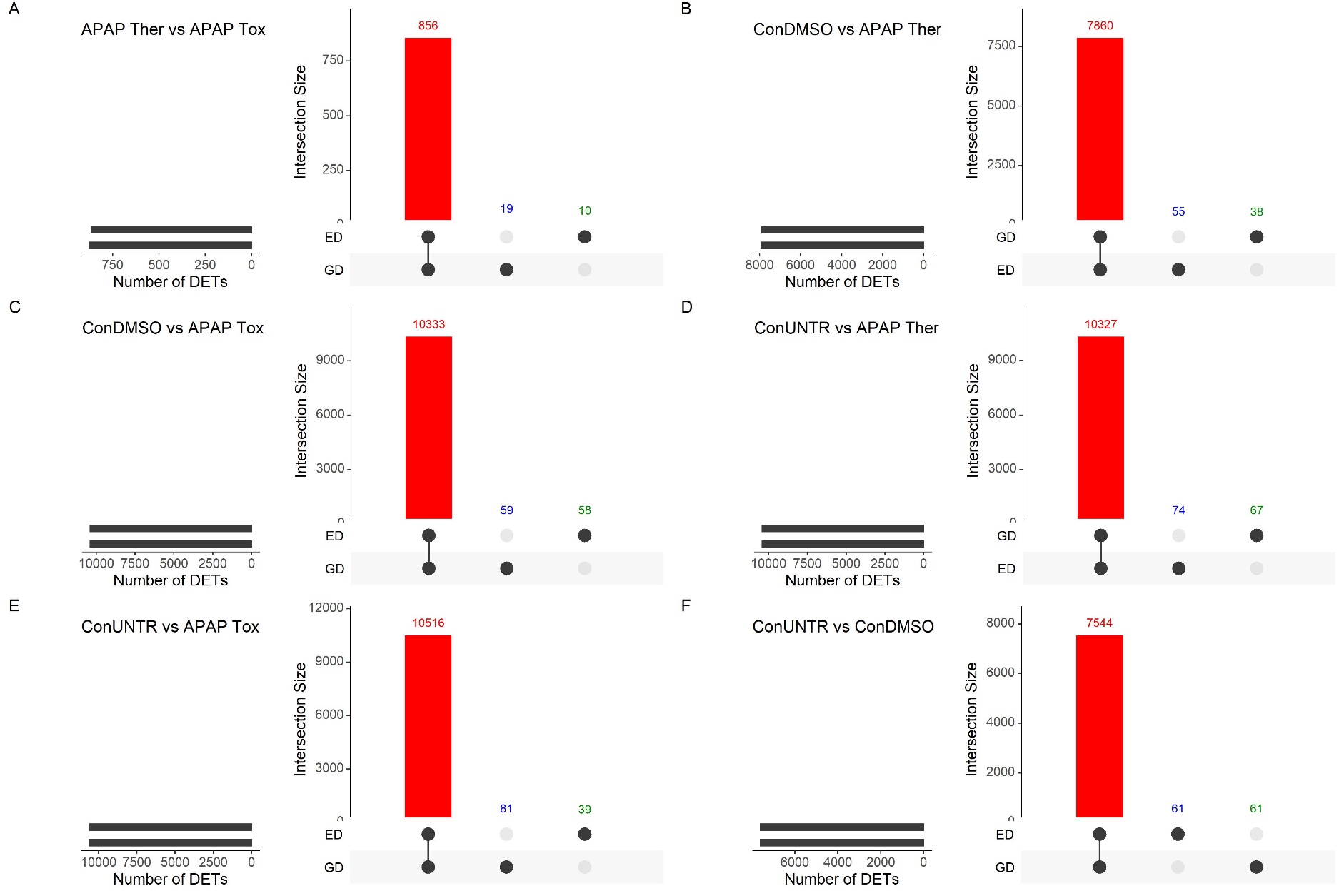


Suppl. Figure 7: Number of DETs and the overlap between them obtained from two SFPG expression calculation methods, namely equal distribution (ED) and group size distribution (GD), available under the “recal_expression” module.

**Suppl. Figures**


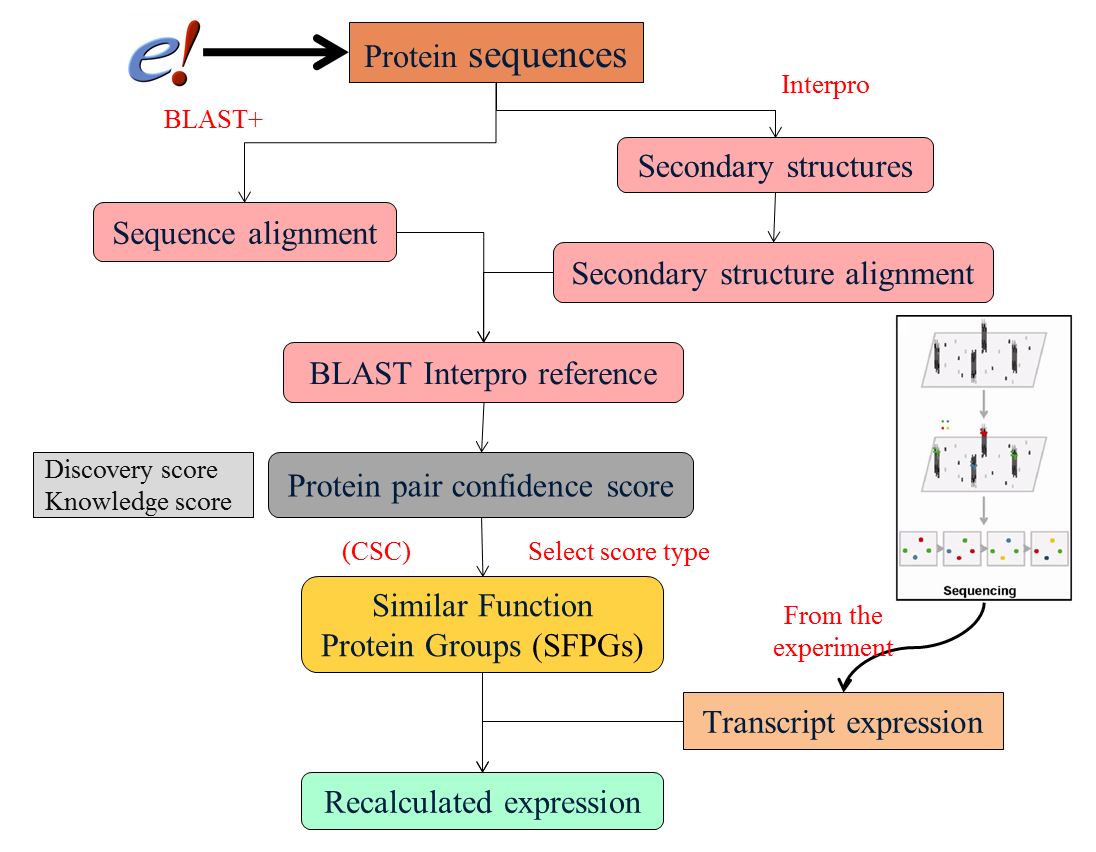
Suppl. Figure 1: FuSe workflow.


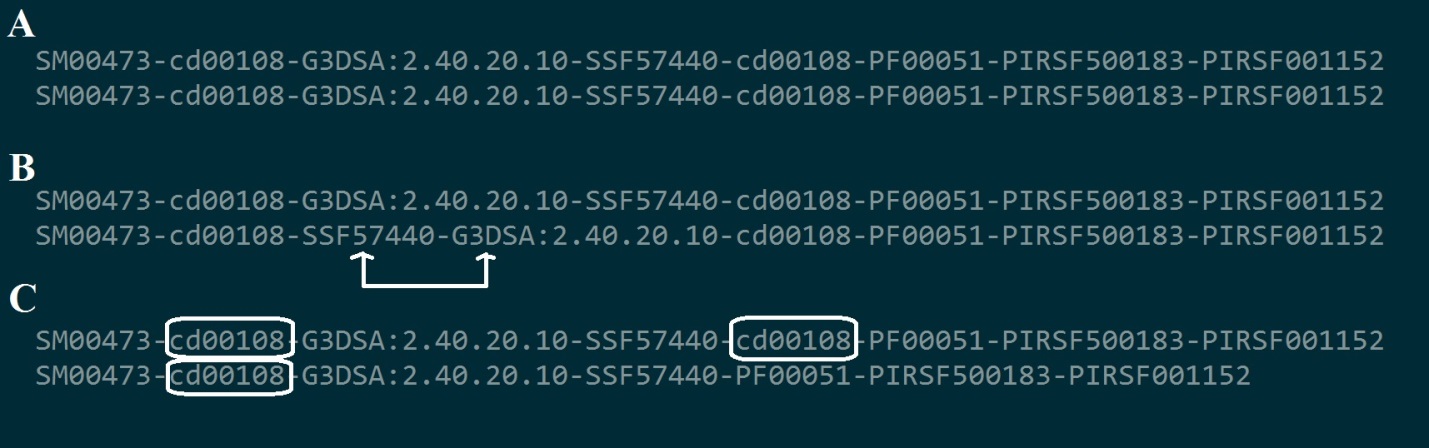


Suppl. Figure 2: Representation of secondary and super secondary structure alignment in FuSe. (A) STONM: same type, order, and number of motifs. (B) STNM: same type and number of motifs. In the illustration, the motifs SSF57440 and G3DSA:2.40.20.10 are interchanged. (C) STM: same type of motifs. In the given illustration the motif, cd00108 has two occurrences in sequence 1 and only one occurrence in sequence 2.


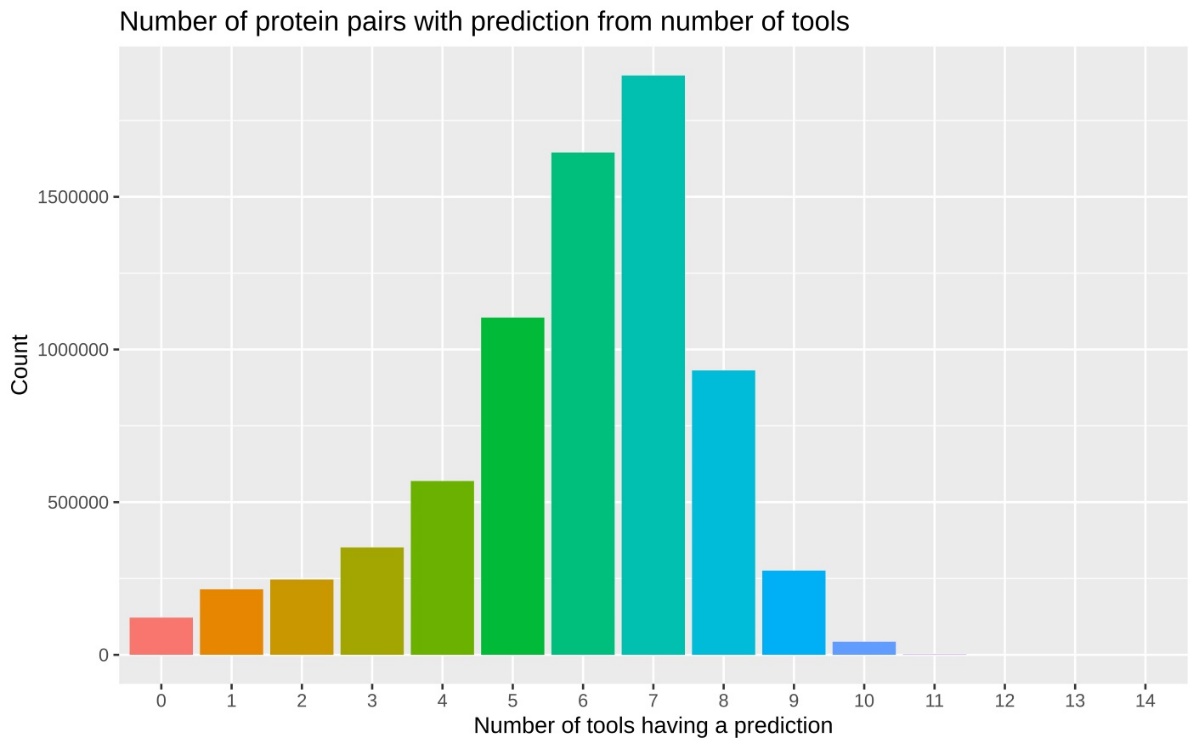


Suppl. Figure 3: The number of protein pairs having a prediction from the Interpro tools. A total of 14 Interpro tools are present, for some proteins the predictions by some tools is not present. When in a protein pair, both the proteins do not have a prediction from a tool, it designated as NP (prediction not present).

Suppl. Figure 4: A dummy example to illustrate the calculation of expression for the SFPGs; the calculations are done based on equation 3.


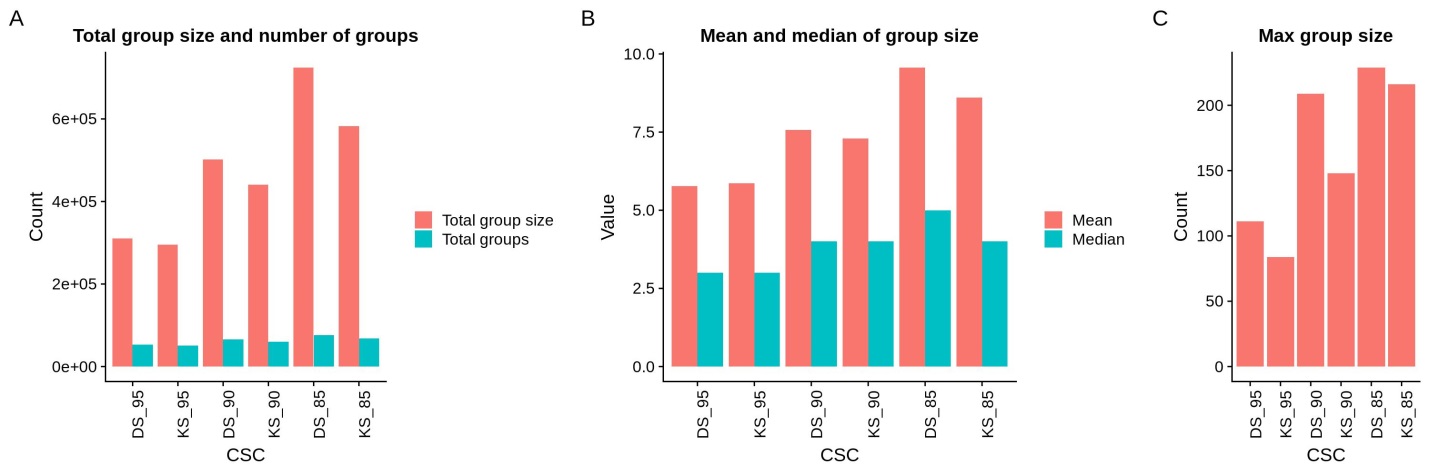


Suppl. Figure 5: Characterization of the SFPGs.


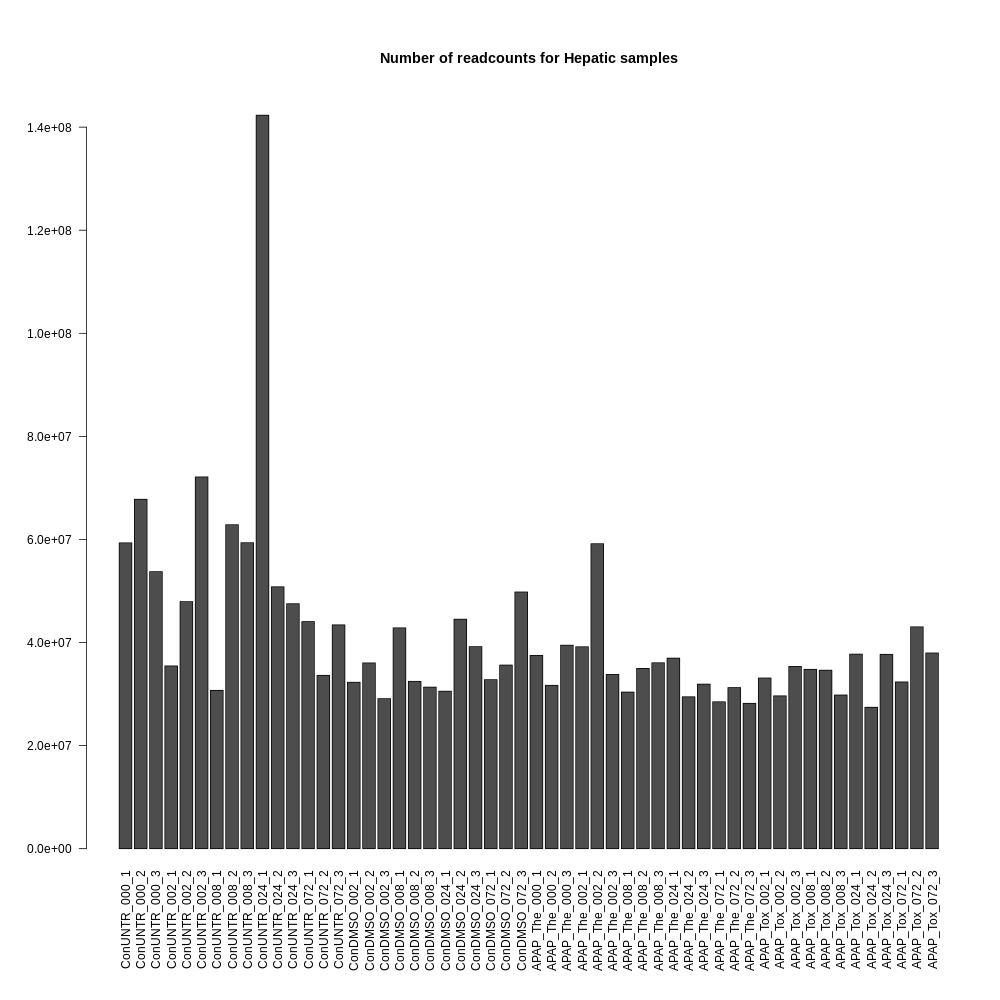


Suppl. Figure 6: Read counts for the samples.

Suppl. Figure7: Number of DETs and overlap between the DETs obtained from two SFPG expression calculation methods available under recal_expression module.

(A)


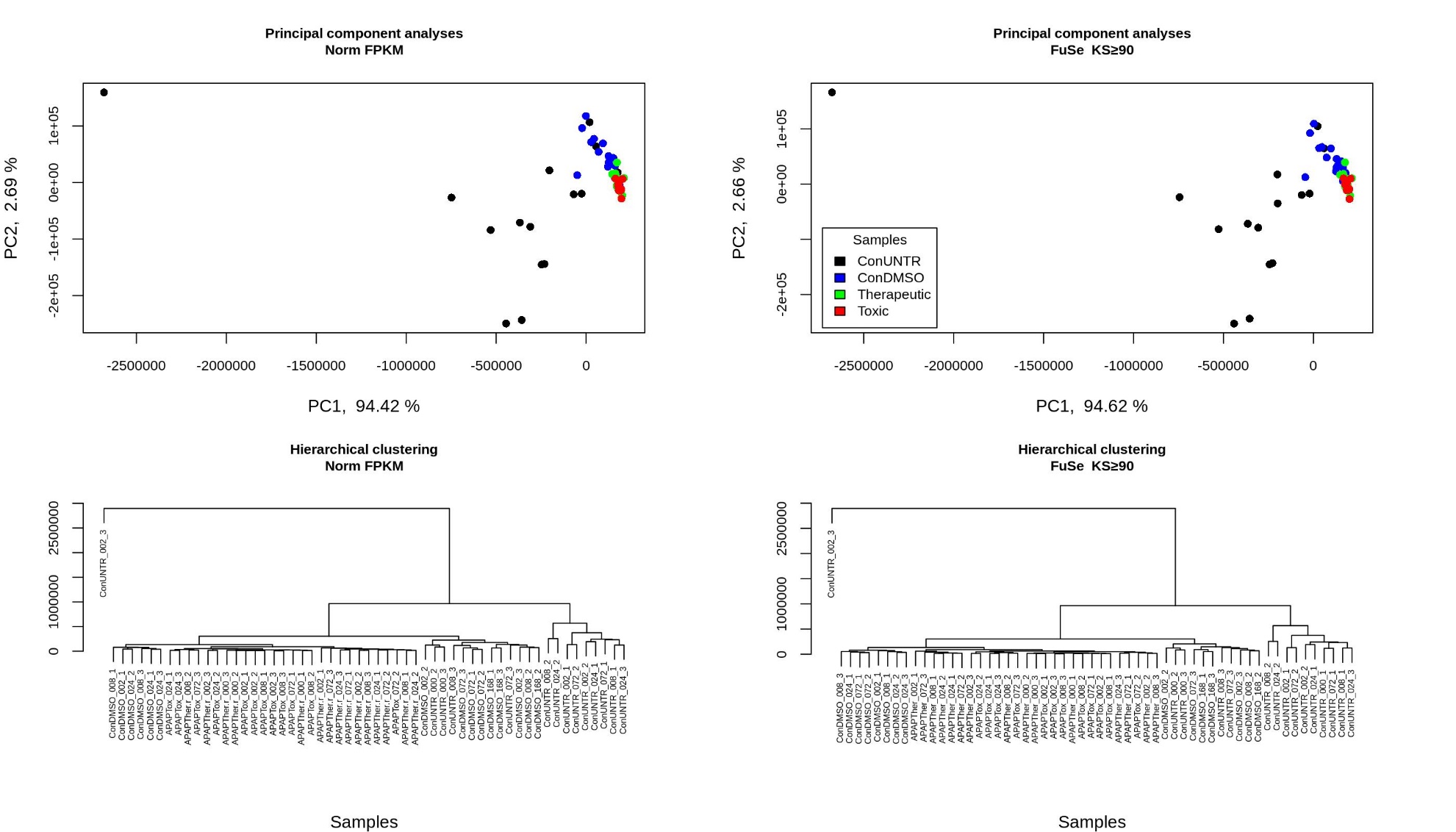


(B)
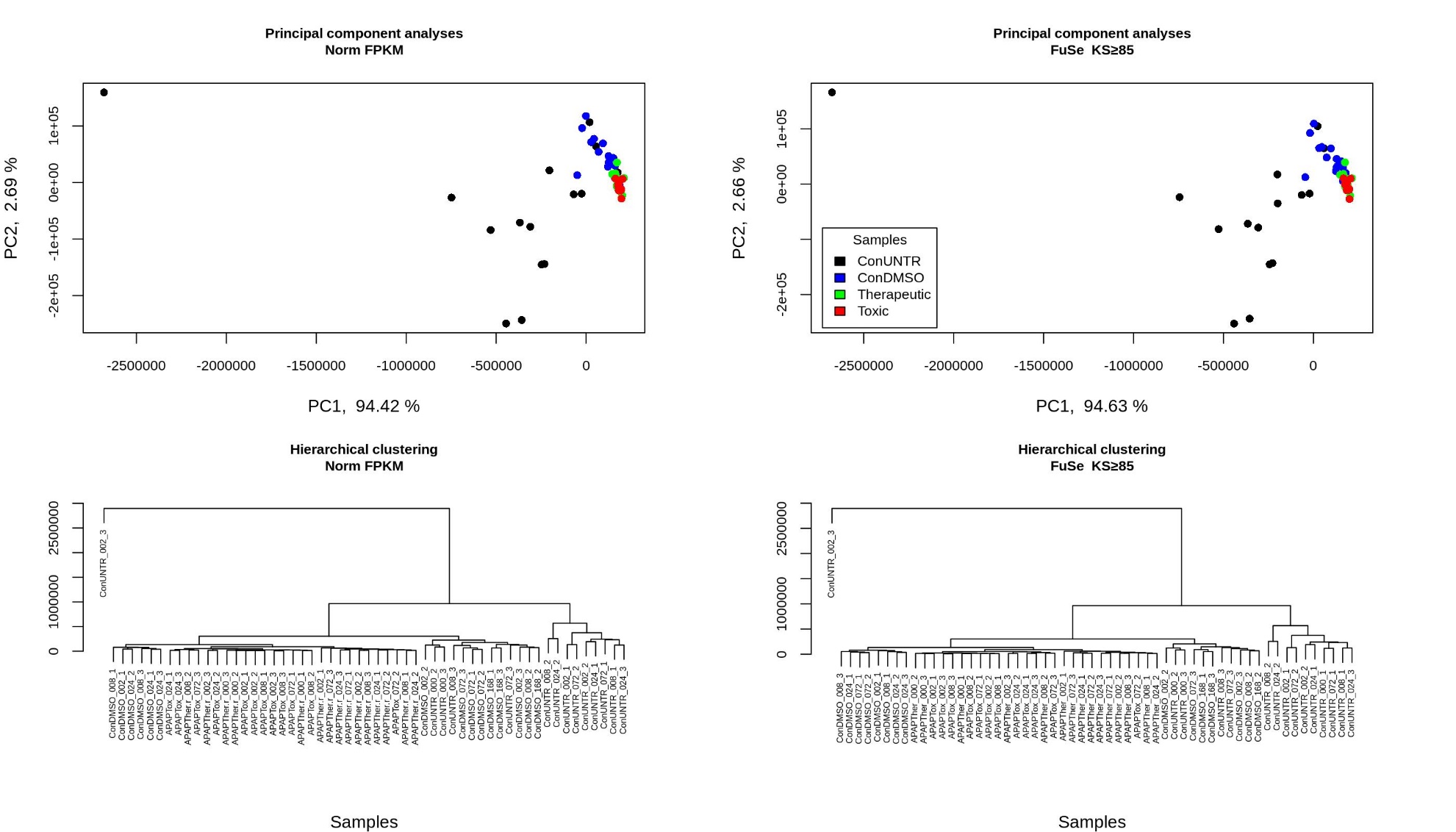


(C)
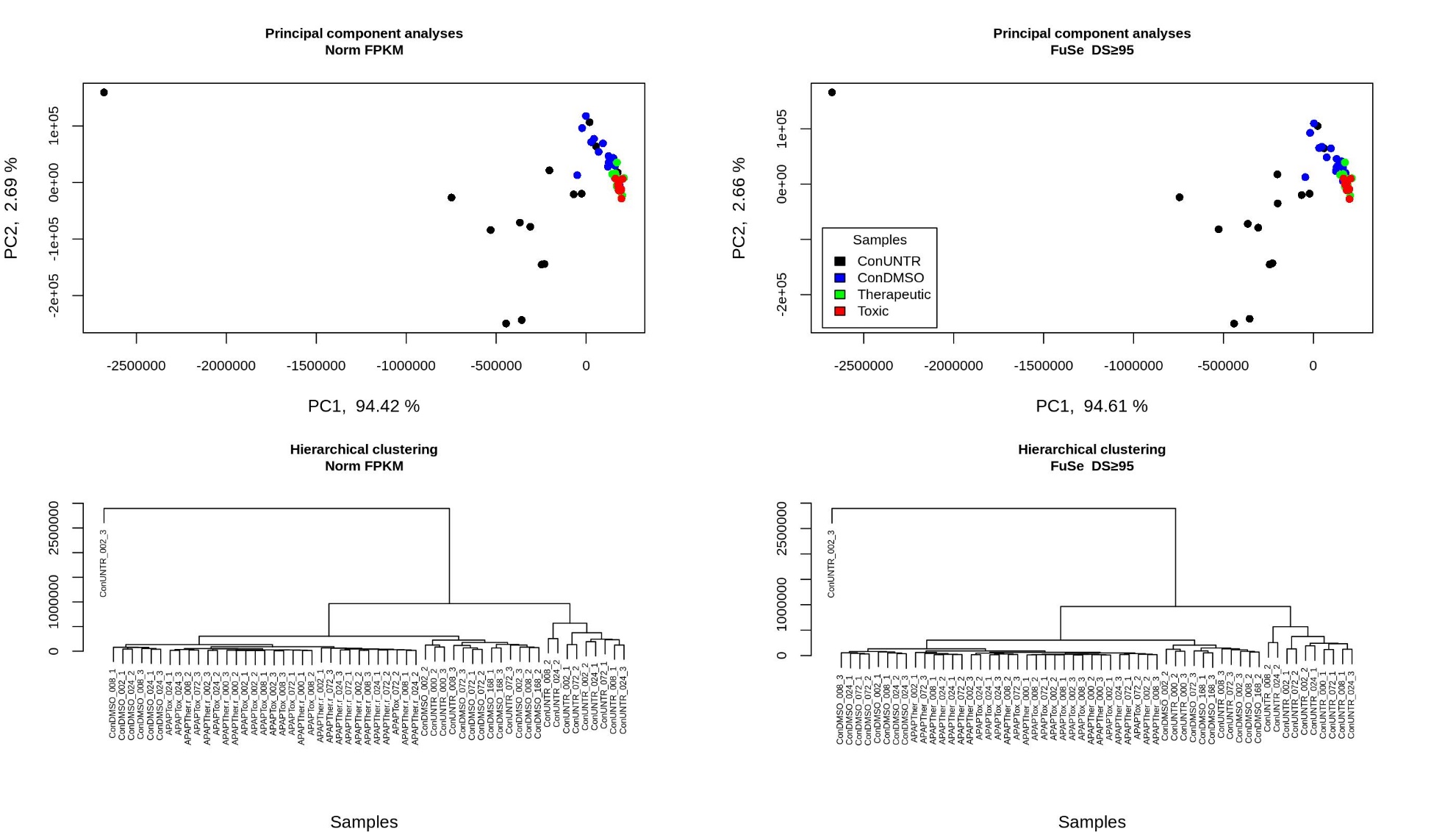


(D)
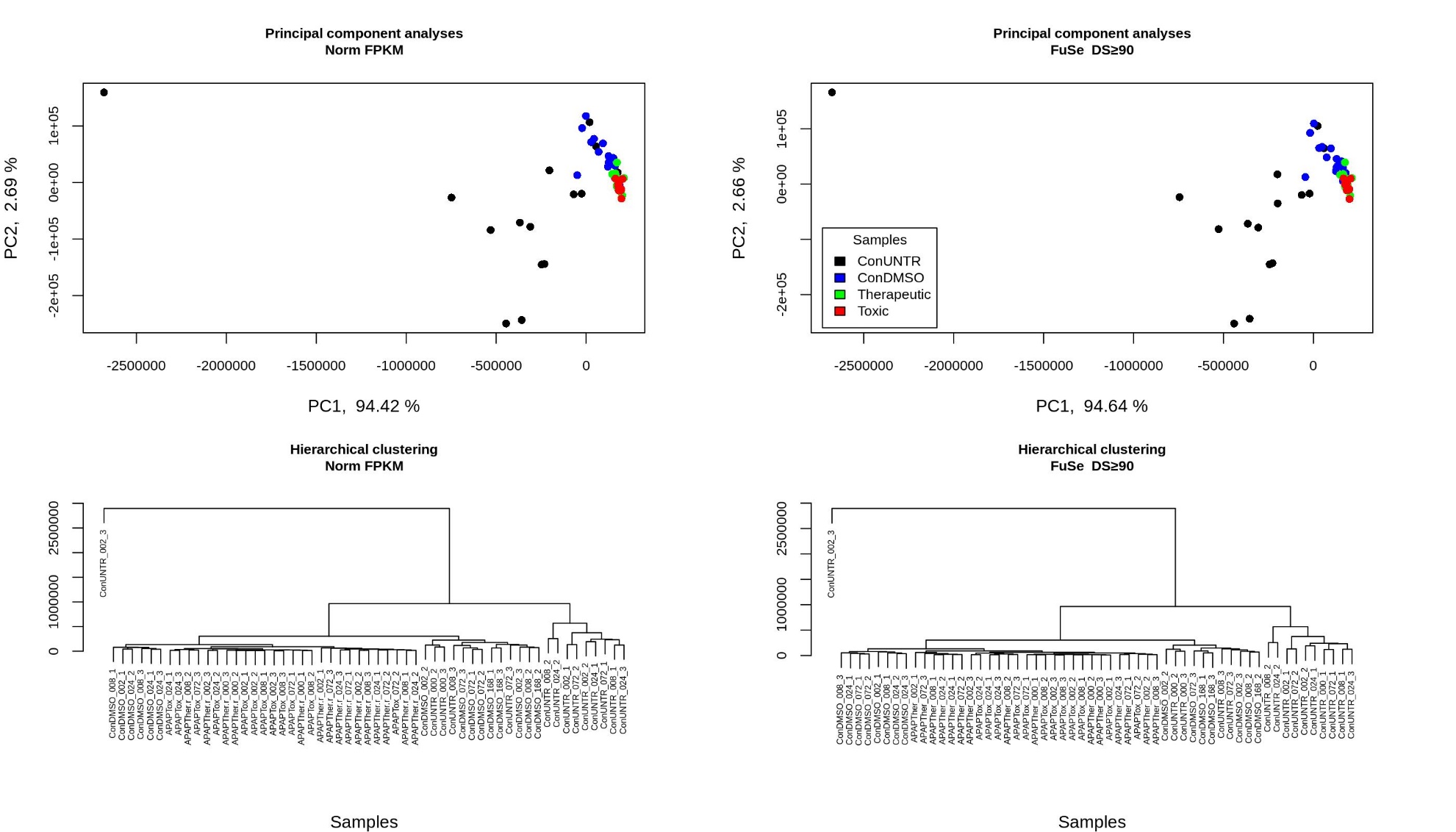
(E)
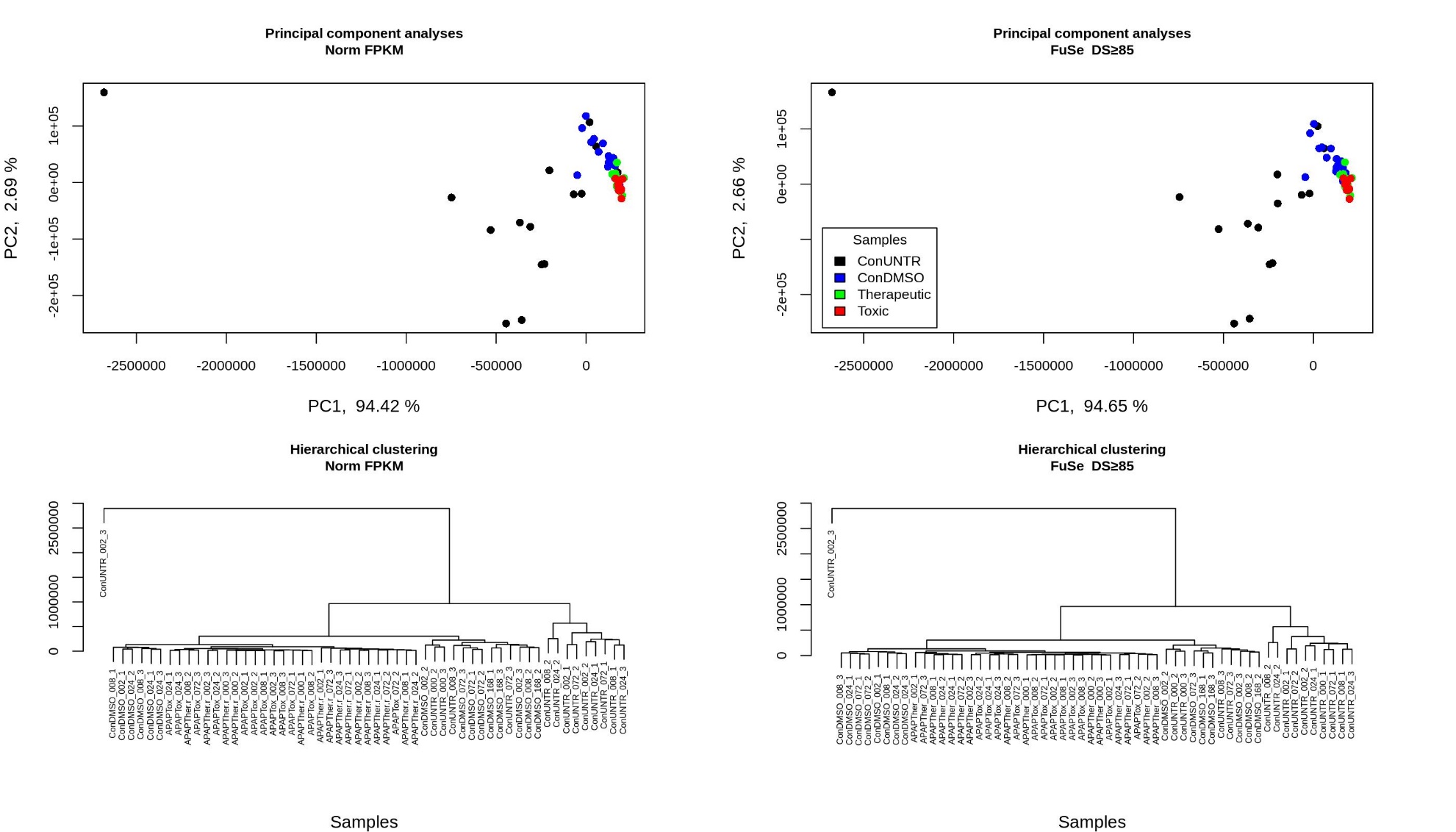


Suppl. Figure 8 (A-E): PCA bi-plots and Hierarchical clustering for the APAP expression data after applying FuSe at different CSC for KS and DS.


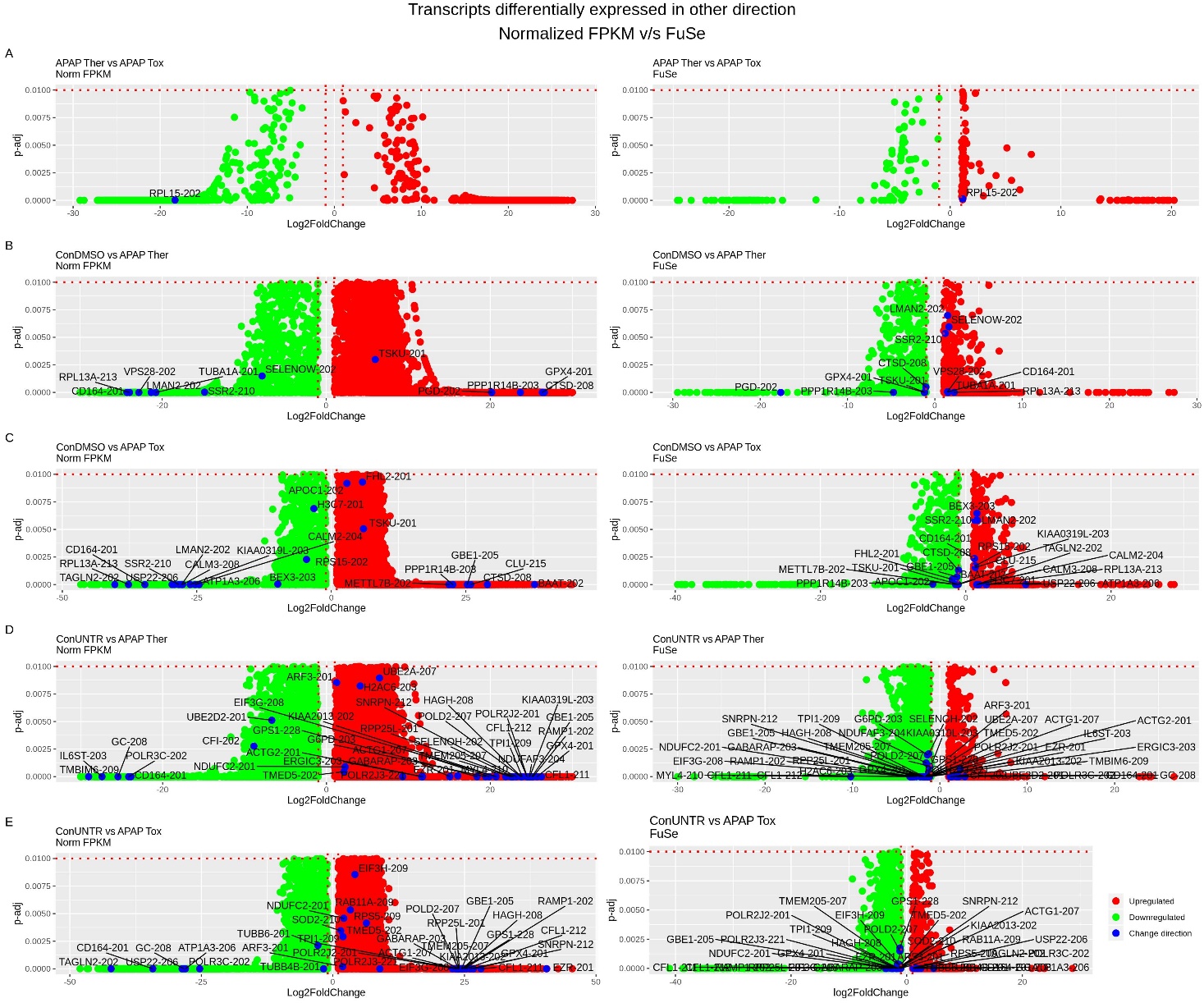


Figure 9: Volcano plot showing the DETs which changed their direction of regulation after analyzing with FuSe.

Supplementary Tables

Suppl. Table 1: DETs computed before (FPKM) and after applying FuSe (SFPG expression; KS>=95) on transcript/isoform expression data from Hepatic cell models exposed to APAP.

References

1. **FastQC A Quality Control tool for High Throughput Sequence Data** [<http://www.bioinformatics.babraham.ac.uk/projects/fastqc/>]

2. Chen S, Zhou Y, Chen Y, Gu J: **fastp: an ultra-fast all-in-one FASTQ preprocessor**. *Bioinformatics* 2018, **34**(17):i884-i890.

3. Ewels P, Magnusson M, Lundin S, Käller M: **MultiQC: summarize analysis results for multiple tools and samples in a single report**. *Bioinformatics* 2016, **32**(19):3047-3048.

4. Bolger AM, Lohse M, Usadel B: **Trimmomatic: a flexible trimmer for Illumina sequence data**. *Bioinformatics* 2014, **30**(15):2114-2120.

5. Aken BL, Ayling S, Barrell D, Clarke L, Curwen V, Fairley S, Fernandez Banet J, Billis K, García Girón C, Hourlier T *et al*: **The Ensembl gene annotation system**. *Database* 2016, **2016**:baw093-baw093.

6. Langmead B, Salzberg SL: **Fast gapped-read alignment with Bowtie 2**. *Nat Methods* 2012, **9**(4):357-359.

7. Li B, Dewey CN: **RSEM: accurate transcript quantification from RNA-Seq data with or without a reference genome**. *BMC Bioinformatics* 2011, **12**:323.

8. Marchler-Bauer A, Lu S, Anderson JB, Chitsaz F, Derbyshire MK, DeWeese-Scott C, Fong JH, Geer LY, Geer RC, Gonzales NR: **CDD: a Conserved Domain Database for the functional annotation of proteins**. *Nucleic Acids Res* 2010, **39**(suppl_1):D225-D229.

9. Lewis TE, Sillitoe I, Dawson N, Lam SD, Clarke T, Lee D, Orengo C, Lees J: **Gene3D: Extensive prediction of globular domains in proteins**. *Nucleic Acids Res* 2017, **46**(D1):D435-D439.

10. Pedruzzi I, Rivoire C, Auchincloss AH, Coudert E, Keller G, de Castro E, Baratin D, Cuche BA, Bougueleret L, Poux S *et al*: **HAMAP in 2015: updates to the protein family classification and annotation system**. *Nucleic Acids Res* 2014, **43**(D1):D1064-D1070.

11. Piovesan D, Tabaro F, Paladin L, Necci M, Mičetić I, Camilloni C, Davey N, Dosztányi Z, Mészáros B, Monzon AM *et al*: **MobiDB 3.0: more annotations for intrinsic disorder, conformational diversity and interactions in proteins**. *Nucleic Acids Res* 2017, **46**(D1):D471-D476.

12. El-Gebali S, Mistry J, Bateman A, Eddy SR, Luciani A, Potter SC, Qureshi M, Richardson LJ, Salazar GA, Smart A *et al*: **The Pfam protein families database in 2019**. *Nucleic Acids Res* 2018, **47**(D1):D427-D432.

13. Wu CH, Nikolskaya A, Huang H, Yeh LSL, Natale DA, Vinayaka CR, Hu ZZ, Mazumder R, Kumar S, Kourtesis P: **PIRSF: family classification system at the Protein Information Resource**. *Nucleic Acids Res* 2004, **32**(suppl_1):D112-D114.

14. Attwood TK: **The PRINTS database: a resource for identification of protein families**. *Briefings in bioinformatics* 2002, **3**(3):252-263.

15. Sigrist CJ, de Castro E, Cerutti L, Cuche BA, Hulo N, Bridge A, Bougueleret L, Xenarios I: **New and continuing developments at PROSITE**. *Nucleic Acids Res* 2013, **41**(Database issue):17.

16. Akiva E, Brown S, Almonacid DE, Barber 2nd AE, Custer AF, Hicks MA, Huang CC, Lauck F, Mashiyama ST, Meng EC: **The structure–function linkage database**. *Nucleic Acids Res* 2013, **42**(D1):D521-D530.

17. Letunic I, Bork P: **20 years of the SMART protein domain annotation resource**. *Nucleic Acids Res* 2017, **46**(D1):D493-D496.

18. Gough J, Karplus K, Hughey R, Chothia C: **Assignment of homology to genome sequences using a library of hidden Markov models that represent all proteins of known structure**. *J Mol Biol* 2001, **313**(4):903-919.

19. Haft DH, Selengut JD, Richter RA, Harkins D, Basu MK, Beck E: **TIGRFAMs and genome properties in 2013**. *Nucleic Acids Res* 2012, **41**(D1):D387-D395.
